# *Caulerpa cylindracea*: First Insight into Its Nutritional Potential

**DOI:** 10.3390/foods14183208

**Published:** 2025-09-15

**Authors:** Neven Iveša, Ines Kovačić, Moira Buršić, Nikola Major, Igor Palčić, Smiljana Goreta Ban, Zoran Užila, Gioconda Millotti

**Affiliations:** 1 Faculty for Natural Sciences, Juraj Dobrila University of Pula, 52100 Pula, Croatia; neven.ivesa@unipu.hr (N.I.); ines.kovacic@unipu.hr (I.K.); moira.bursic@unipu.hr (M.B.); 2 Department of Agriculture and Nutrition, Institute of Agriculture and Tourism, Carlo Hugues 8, 52440 Poreč, Croatia; nikola@iptpo.hr (N.M.); palcic@iptpo.hr (I.P.); smilja@iptpo.hr (S.G.B.); zoran@iptpo.hr (Z.U.)

**Keywords:** *Caulerpa cylindracea*, proximal composition, minerals, vitamins, amino acids, fatty acids

## Abstract

The invasive seaweed *Caulerpa cylindracea* is widespread in the Mediterranean and has notable ecological impacts, yet its nutritional potential remains underexplored. This study aimed to characterize the nutritional composition of *C. cylindracea* comprehensively. Samples were collected from the Northern Adriatic and analyzed for proximate composition, amino acids, minerals, vitamins, and fatty acids using standardized laboratory methods. The results revealed a balanced proximate profile with notable protein (11.8 g/100 g DW) and fiber (24.4 g/100 g DW) levels and relatively low carbohydrates (11.6 g/100 g DW). The seaweed exhibited a rich mineral content, including high levels of iron, magnesium, manganese, and potassium, while toxic heavy metals were absent. Vitamins B12 and E were present at elevated concentrations compared to related species. Amino acid analysis showed a well-balanced essential amino acid profile supporting its nutritional value. The high salt content (33.8 g/100 g DW) suggests the need for desalination prior to consumption to reduce sodium intake risks. These results indicate the potential of this invasive species as a novel dietary component, particularly for populations who may benefit from plant-based marine sources of essential nutrients in the Mediterranean region. This is the first comprehensive nutritional characterization of *C. cylindracea* from the Adriatic Sea, highlighting its potential for valorization as both an environmental management strategy and a novel dietary resource.

## 1. Introduction

The genus *Caulerpa* is among the most widely distributed genera worldwide [1]. Algae belonging to the *Caulerpa* family are collectively known as “sea grapes” and are already exploited for human consumption [2,3,4]. *Caulerpa cylindracea* is a well-known alien seaweed in the Mediterranean Sea due to its invasive properties [5]. It grows rapidly, invades various habitats, and spreads through fragmentation and propagation, leading to significant structural and functional changes in marine communities and impacting fish metabolism [6].

Since its first detection in Croatian waters in the year 2000 in the southern Adriatic [7], it rapidly spread over the following six years toward the shallow, highly productive northern part of the Adriatic, where it colonized various substrates, including shallow infralittoral zones with photophilic macroalgae, seagrass meadows, and areas of mobile substrate with sand and mud [8,9]. Preliminary field surveys of the infralittoral zone using scuba diving equipment in the southern Istria region indicate that *C. cylindracea* is present on parts of the mobile substrate at depths of 10 to 20 m, which is particularly prominent in the Medulin Bay area, where it covers surfaces spanning several square kilometers [10]. A study conducted in Funtana Bay investigated the effects of *C. cylindracea* on the seagrass *Cymodocea nodosa* and demonstrated that the extensive proliferation of this invasive alga significantly reduced the below-ground biomass of *C. nodosa*, which plays a critical role in sediment stabilization and anchorage [11]. This decline consequently disrupted the stability of the entire seagrass meadow, which otherwise plays an important ecological role in coastal ecosystems—ranging from preventing erosion to providing habitats for numerous marine organisms.

The direct consumption of sea algae as a primary food source remains uncommon in the Adriatic Sea and in the Mediterranean [12]. Macroalgal production faces significant obstacles, including the absence of tradition, high production costs, and the underdevelopment of local supply chains. Additional challenges arise from the lack of national strategies and incentives to support enterprises and limited knowledge regarding the nutritional properties of macroalgae. Given the pronounced adaptability of *C. cylindracea* to local anthropogenic pressures and its broader resilience to climate change, coupled with its rapid growth—characteristic of many invasive non-native algal species—this alga may represent a viable feedstock for industrial applications [13]. Studying the biochemical composition of *C. cylindracea* in the Adriatic context is, therefore, of high importance, as it would provide the first insight into whether this abundant biomass could represent an alternative source of nutrients. This question gains further relevance under the combined pressures of global warming and increasingly challenging agricultural conditions, which are pushing the search for sustainable and climate-resilient food sources [14,15].

A review analyzing the state of seaweed bio-based products and research in the Mediterranean over the past 20 years indicates that, despite the region’s rich marine biodiversity, seaweed utilization, particularly for human consumption, remains limited [16]. As agriculture faces growing challenges due to crop diseases and climate change, seaweed could serve as a sustainable alternative in nutrition. Seaweeds are a promising option as they produce abundant protein with a low carbon footprint [17]. Moreover, with the increasing popularity of vegan diets, there is a need to expand the variety of locally available foods that cater to this dietary preference. Seaweed is considered a valuable source of plant-based protein, especially for individuals following vegetarian or vegan diets, as it provides essential amino acids necessary for human health [18].

*C. cylindracea* has been reported to have high palatability, which aligns with its low levels of the toxic and feeding-deterrent sesquiterpene caulerpynene [3,19]. In contrast, this compound is abundant in the congeneric, inedible *Caulerpa taxifolia* [3]. Moreover, *C. cylindracea* contains significantly higher levels of the harmless bisindole alkaloid caulerpin (CAU) [20], which exhibits a range of biological activities relevant to food science and pharmacology [3,21]. The nutritional composition of various *Caulerpa* species is already known, either in full or in part. Examples include *Caulerpa racemosa* var. *cornyphosa*, *Caulerpa racemosa* var. *macrophysa*, *Caulerpa occidentalis*, *Caulerpa scalpelliformis* [22], *Caulerpa lentillifera* [23,24,25], and *Caulerpa racemosa* [4].

Previous work by Tahar et al. [26] reported some aspects on the nutritional composition of *C. cylindracea* collected from the Mediterranean Sea, including its total lipid, protein, and ash profile, as well as its fatty acid and bioactive compound contents. However, due to the pronounced regional variability in environmental conditions and their influence on nutritional composition, it is essential to investigate additional populations of *C. cylindracea* from both the Adriatic and Mediterranean regions. Importantly, there are currently no data on its vitamin content, detailed amino acid profile, or mineral composition. Understanding its nutritional profile could, therefore, highlight its possible role as a novel dietary component, especially for vegan and vegetarian populations who benefit from plant-based marine sources of essential nutrients. To our knowledge, the only other nutritional data available in the literature for *C. cylindracea* is the fatty acid profile reported by Blažina et al. in 2009 [27].

This study provides, for the first time in the Adriatic region, a comprehensive nutritional characterization of *C. cylindracea*, including proximate composition, minerals, vitamins, amino acids, and confirmation of its fatty acid profile. Our findings highlight the potential of this invasive seaweed as a sustainable dietary resource and as a strategy for environmental management in the Mediterranean.

## 2. Materials and Methods

### 2.1. Seaweed Harvest and Preparation

**Figure 1:**
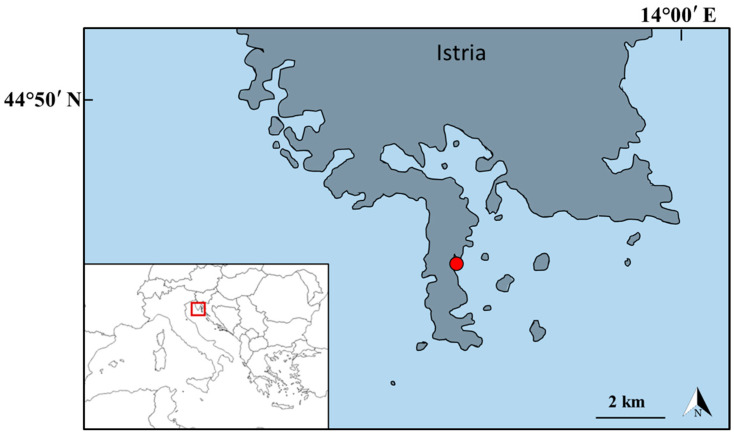
Sampling location (marked with a red dot) in the northern Adriatic Sea (44°47′25.2″ N 13°55′02.8″ E).

Samples of *Caulerpa cylindracea* were collected in December 2024 from the infralittoral area of the western coast of the Bay of Medulin in the proximity of the protected area of Lower Kamenjak and Medulin archipelago at an average depth of 12 m (Figure 1). According to the Köppen climate zones, this area corresponds to a humid temperate climate with hot summer (Cfa) [28].

The collected seaweed was soaked and afterwards washed thoroughly with running water to eliminate epibionts and sediment. The seaweed was dried in a food dehydrator (manufactured by Gorenje, model FDK24DW) at 35 °C for 18 h. Afterwards, they were stored in a vacuum container in the dark until use.

### 2.2. Determination of Proximal Nutritional Values

The nutritional composition of the dried seaweed was determined in collaboration with Laboratory Group Ltd., J.S. Hamilton Poland, Gdynia, Poland, an accredited laboratory certified in accordance with international standards and requirements. The energy value was determined according to Regulation (EU) No 1169/2011. The total protein was determined with PB-116 ed. III of 11 August 2020, with the Kjeldahl method. Carbohydrates were determined according to Regulation (EU) No 1169/2011 (sum 100%); sugars with PB-429 ed 3 of 29 November 2024 were determined with ion chromatography with pulsed amperometric detection; fibers with AOAC 991.43:1994; lipids by PB-286 ed. 2 of 16 January 2025 with Soxhlet extraction and weight determination; and saturated fatty acids by PN-EN ISO 12966-1:2015-01, PN-EN ISO 19226-2:2017-05, except p. 5.3 and 5.5, and PN-EN ISO 12966-4:2015-07 with gas chromatography with a flame ionization detector (GC-FID). Moisture was determined by PN-ISO 1026:2000, drying the sample at 70 °C under reduced pressure and weight loss determination. Ash was determined by PN-A-75101-081990; PN-a-75101-08:1990/Az1:2002, consisting of incineration of the sample at 525 °C ± 25 °C and weight determination of the ash content. Salt (NaCl) was determined by PB-318 ed. 3 of 11 October 2024, using flame atomic absorption spectroscopy (FAAS), inductive coupled plasma mass spectroscopy (ICP-MS), and inductively coupled plasma optical emission spectrometry (ICP-OES).

### 2.3. Determination of Amino Acid Content

The amino acid content of the dried seaweed was determined in collaboration with Laboratory Group Ltd., J.S. Hamilton Poland, an accredited laboratory certified in accordance with international standards and requirements, following the method PB-53/HPLC ed. II 30 December 2008 using pre-column HPLC-UV, while tryptophan determination was carried out according to PB-136/HPLC ed. I of 6 February 2012 with an HPLC with fluorometric detection.

### 2.4. Determination of Fatty Acid Content

Fatty acid composition of the dried seaweed was determined in collaboration with Laboratory Group Ltd., J.S. Hamilton Poland, an accredited laboratory certified in accordance with international standards and requirements following the standards PN-EN ISO 12966-1:2015-01, PN-EN ISO 12966-2:2017-05 (excluding p. 5.3 and 5.5), and PN-EN ISO 12966-4:2015-07. The analysis of fatty acid was carried out by GC-FID. The results were expressed as mg/100 g of algae dry weight.

### 2.5. Determination of Mineral Content

The determination of macro and micro elements (Al, B, Ca, Cd, Co, Cr, Cu, Fe, K, Li, Mg, Mn, Mo, Na, Ni, P, Pb, S, Se, Si, and Zn) was carried out with inductively coupled plasma–optical emission spectroscopy, ICP-OES (ICPE-9800 Shimadzu, Kyoto, Japan), after microwave-assisted digestion (Ethos Up, Milestone, Sorisole, Italy). Briefly, 200 mg of the oven-dried sample was digested with 6 mL of concentrated HNO_3_ and 2 mL of 30% H_2_O_2_, transferred to a 25 mL volumetric flask, and filled to the mark with ultrapure water. The samples were stored at 4 °C until analysis. Single-element standard solutions (Inorganic Ventures, Christiansburg, VA, USA) were used in order to control the plasma positioning and preparation of calibration standard solutions. The calibration standard was prepared by serial dilution of a stock solution (concentration range from 0.01 to 15 mg/L). Calibration curves were fitted with linear regression (R^2^ > 0.999). The method accuracy evaluation was carried out using certified reference material (WEPAL, Wageningen, The Netherlands). Trace elements were measured axially, while major elements were measured radially to avoid saturation. Operating parameters were as follows: 1.15 kW of RF power, 12 L/min of a plasma flow rate, 0.5 L/min of an auxiliary gas flow rate, and 0.5 L/min of a nebulizer flow rate. Sample solutions were introduced into the plasma using a concentric nebulizer and a cyclonic-type spray chamber. Argon (99.999% pure, Linde Gases, Ananindeua, PA, Brazil) was used to purge the optics and to form the plasma. Limits of detection and quantification can be found in Table S1.

### 2.6. Determination of Vitamin Content

Vitamin concentrations were analyzed using an LC-MS/MS system comprising an autosampler (Shimadzu Nexera SIL-40CX3, Kyoto, Japan), two solvent delivery modules (Shimadzu Nexera LC-40DX3, Kyoto, Japan), a column oven (Shimadzu Nexera CTO-40C, Kyoto, Japan), and a triple–quadrupole mass spectrometer (Shimadzu LCMS8045, Kyoto, Japan). For sample preparation, 250 mg of dried material was homogenized in 10 mL of 80% aqueous methanol using a benchtop vortex mixer (Biosan V-1, Riga, Latvia) for 2 min. This was followed by maceration on a rotator (Biosan RS-60, Riga, Latvia) for 20 h at 25 °C. The extract was then centrifuged for 10 min at 4000× *g* (Tehtnica Centric 350, Podplat, Slovenia) and filtered through a 0.22 µm nylon membrane. Chromatographic separation was performed on a Discovery^®^ HS F5-3 column (2.1 mm × 150 mm, 3 µm core–shell; Sigma-Aldrich, St. Louis, MO, USA) maintained at 37 °C. Then, 1 µL of aliquot was injected and separation was achieved using a linear gradient between mobile phase A (water with 0.1% formic acid) and mobile phase B (methanol with 0.1% formic acid), at a flow rate of 0.25 mL/min. The gradient profile was as follows: 0–2 min, 100% A; 2–15 min, gradient from 100% A to 5% A; 15–20 min, 5% A; 20–20.1 min, return to 100% A; and 20.1–25 min, 100% A. Target compounds were identified and quantified by matching retention times, characteristic precursor/product ion transitions, and peak areas to those of authentic standards. Vitamin B12 content of the dry seaweeds was determined in collaboration with Laboratory Group Ltd., J.S. Hamilton Poland, an accredited laboratory certified in accordance with international standards and requirements. Vitamin B12 was determined by the method PB-328 ed. 2 of 5 September 2022 and consisted of microbiological determination using a strain of *Lactobacillus leishmannii*.

### 2.7. Data Analysis

All analyses were performed in triplicate from each sample (n = 3) and results are expressed as average ± SD. The data were analyzed using Statistica 10.0 (StatSoft, Inc., Tulsa, OK, USA).

## 3. Results

### 3.1. Proximate Composition

The proximate composition reveals a relatively low carbohydrate content of 11.6 g/100 g dry weight (DW). Although the carbohydrate content in this case is on the lower end, it is particularly noteworthy that the carbohydrate and protein values are nearly equal—an uncommon occurrence, as carbohydrate levels in seaweed are typically much higher than protein levels. The fiber content of *C. cylindracea* was 24.4 g/100 g DW. The seaweed’s salt content of 33.8 g/100 g DW is notably high, making it less suitable for direct consumption due to the risk of an excessive sodium intake. The results of the proximate composition are summarized in Table 1.

### 3.2. Protein Content and Amino Acids

The protein content of *Caulerpa cylindracea* was found to be 11.8 g/100 g of DW. The total essential amino acid (EAA) content is 3362 mg/100 g DW, while non-essential amino acids (NEAAs) amount to 3860 mg/100 g DW. This results in an EAA/NEAA ratio of 0.87, which reflects a well-balanced amino acid composition. Among the amino acids, glutamic acid (630 mg/100 g DW) and leucine (610 mg/100 g DW) are the most abundant non-essential and essential amino acids, respectively. The amino acid composition expressed in mg/100 g DW seaweed is expressed in Table 2.

### 3.3. Mineral Content

The mineral content in *C. cylindracea* is particularly noteworthy. As previously mentioned, the sodium concentration is significantly higher than nearly all values reported for *C. racemosa*. The potassium (K) level is approximately 2-fold higher than the average values for *C. racemosa*. Calcium, zinc, and copper levels are around the average values of *C. racemosa*. Interestingly, magnesium and iron concentrations are well above the average content in *C. racemosa*, and the manganese content in *C. cylindracea* is approximately four times higher than any values reported for *C. racemosa*. Notably, potentially harmful elements such as cadmium (Cd), cobalt (Co), nickel (Ni), and chromium (Cr) were not detected. The mineral content of *C. cylindracea* is summarized in Table 3.

### 3.4. Vitamin Content

The vitamin profile of *C. cylindracea* revealed the presence of several B-complex vitamins and a notably high content of vitamin E (Table 4). Among the B vitamins, nicotinic acid (B3) was the most abundant (5.14 µg/g DW), followed by niacinamide (1.53 µg/g DW), pantothenic acid (1.43 µg/g DW), and riboflavin (0.996 µg/g DW). Lower levels were detected for pyridoxal (0.59 µg/g DW), folic acid (0.060 µg/g DW), pyridoxine (0.092 µg/g DW), biotin (0.031 µg/g DW), and cyanocobalamin (vitamin B12, 0.39 µg/g DW). Notably, the concentration of vitamin E (expressed as alpha-tocopherol) reached 525 µg/g DW, significantly exceeding values typically reported in other *Caulerpa* species.

### 3.5. Fatty Acid Content

The total fat content in the analyzed seaweed was 13 mg/g DW (1.3 g/100 g DW). A fatty acid analysis revealed that saturated fatty acids (SFA) accounted for 4 (1 ± 1) mg/g DW of seaweed, with palmitic acid (C16:0) being the most abundant individual SFA at 3 (1 ± 1) mg/g DW of seaweed. Other saturated fatty acids were present in trace amounts (<1 mg/g DW). Monounsaturated fatty acids (MUFA) totaled 3 (1 ± 1) mg/g DW, dominated by oleic acid (C18:1n9) at 2 (1 ± 1) mg/g DW. Other MUFAs were also detected only in trace amounts. Polyunsaturated fatty acids (PUFA) comprised the largest group, representing 5 (1 ± 1) mg/g DW. The main PUFAs identified were α-linolenic acid (C18:3n-3, ALA) at 2 (1 ± 1) mg/g of seaweed, arachidonic acid (C20:4n6), and eicosapentaenoic acid (C20:5n3, EPA), both at 1 (1 ± 1) mg/g DW. The total omega-3 fatty acid content was 3 (1 ± 1) mg/g DW, omega-6 fatty acids accounted for 1 (1 ± 1) mg/g DW, and omega-9 fatty acids made up 2 (1 ± 1) mg/g DW. Trans-fatty acids measured below 1 (1 ± 1) mg/g DW (Table 5). Overall, the seaweed exhibited a low total fat content with a fatty acid profile rich in polyunsaturated fatty acids, particularly omega-3 fatty acids, which are considered beneficial for human nutrition.

## 4. Discussion

Climate change, along with the depletion of land and freshwater resources, is driving both populations and scientists to explore alternative sources of food and nutrition beyond traditional methods [29]. Among these alternatives, seaweeds have garnered significant attention in recent years and are often referred to as the “food of the future” [30]. The nutritional potential of seaweeds has gained increasing recognition, not only due to climate pressures and resource limitations, but also because of their distinctive biochemical profiles. Within green algae, species of the genera *Ulva* and *Caulerpa* stand out, as many are directly incorporated into human diets, underscoring their growing relevance as sustainable food resources.

In particular, *Caulerpa cylindracea* has attracted interest for its moderate protein content (≈8–10% DW) and relatively low lipid fraction (~2% DW), combined with a remarkably high proportion of polyunsaturated fatty acids (PUFA), frequently exceeding 40% of total fatty acids [26,31]. Similar nutritional characteristics are observed in other edible green species such as *C. lentillifera* and *Ulva* spp., which are also rich in minerals and essential fatty acids [32]. In contrast, brown and red algae, including *Ascophyllum nodosum* and *Palmaria palmata f. mollis*, generally provide lower protein levels, but are particularly valued for their bioactive compounds, dietary fibers, and broad applications in medicine, pharmacology, and agronomy [11,33,34]. These functional distinctions highlight the specific role of green algae—and especially, *C. cylindracea*—in the development of future sustainable food systems. *C. cylindracea* is one of the most successful non-indigenous species (NIS) in the Mediterranean [35]. Over the last decade, key policies have been implemented to manage invasive NIS in Mediterranean EU and non-EU countries [36]. However, many NIS are well-established, eradication is costly and uncertain, and the EU discourages excessively expensive measures [37]. Since complete eradication is impractical, adaptive management is crucial [38]. Notably, some NIS could potentially offer benefits [35]—here, the potential of *C. cylindracea* as a food resource is explored for the first time based on its nutritional value.

As this is the first time *C. cylindracea* is being studied for its nutritional composition, a direct comparison with existing data is not possible. Therefore, the values obtained were compared with the most extensively studied *Caulerpa* species, such as *C. racemosa*. It is important to note that even within a single species—for example, *C. racemosa*—there is significant variation in nutritional values depending on the sampling location. This suggests that comparisons are inherently difficult and that *C. cylindracea* may also exhibit variation in its nutritional composition based on where and when it is collected.

The naturally high salt concentration of this seaweed—measured at 33.8 g/100 g DW—raises important concerns for its use as a food ingredient. Such a high sodium content was also reported for *C. racemosa* (21.9%) by Paul et al. [2]. While seaweeds are widely appreciated for their nutritional value, including their content of minerals, vitamins, and dietary fiber, excessive sodium levels limit their suitability for direct consumption. This is particularly critical for individuals on salt-restricted diets, including those with hypertension or kidney-related conditions, for whom a high sodium intake could pose serious health risks [39]. To address this issue and improve the seaweed’s nutritional acceptability and sensory appeal, a range of desalination techniques and post-desalination processes can be employed [40,41]. For example, Park et al. [41] evaluated the use of *Codium fragile* (CF) as a functional food and recorded a salt content of 39.8% in the dried CF. Rinsing under running water reduced the salt content to 18.8%. Further desalination through water immersion—optimized for the temperature and immersion time—followed by post-desalination treatments (including sonication, vacuum pulsing, and centrifugal dehydration) reduced the salt content to as low as 0.7% after centrifugal dehydration. The careful optimization of these treatments is essential to minimize the sodium content without compromising the health-promoting bioactive components such as polyphenols and sulfated polysaccharides, which are responsible for many of seaweed’s antioxidant, anti-inflammatory, and gut health benefits [42]. In this regard, the results reported by Park et al. [41] demonstrated that the desalination of raw CF before the drying process not only removed salt, but also enhanced the phenol-based functionality. These findings suggest that optimal desalination conditions, which result in the lowest salt content, may also contribute to an increase in the total phenolic content. A similar effect was observed in desalinated *Acanthus ebracteatus* Vahl. extracts, which demonstrated increased anticancer potential [43]. Therefore, the use of *C. cylindracea* in larger quantities remains feasible, provided it undergoes an appropriate desalination process.

Previous studies have reported that *C. racemosa*’s carbohydrate content can vary widely, ranging from approximately 30% to 89% DW [4,44]. These variations are influenced by factors such as the season, temperature, and geographical location [4]. The obtained carbohydrate content of 11.6 g/100 g DW is significantly lower than the values reported in the literature for *C. racemosa* samples. For example, Robledo et al. [45] and Kumar et al. [46] both reported carbohydrate levels of 48.95 g/100 g DW, while Renaud et al. [47] and Rameshkumar et al. [48] recorded even higher values of 83.2 g/100 g. Additional studies, such as those by Hong et al. [49] and Bhuylan et al. [50], reported values of 75.3 g/100 g and 48.97 ± 1.22 g/100 g, respectively. Even the lowest reference values, such as 31.2 ± 4.9 g/100 g [51] and 33.42 ± 1.34 g/100 g [14], are almost three times higher than the carbohydrate level found in the current sample. Such a deviation suggests either a unique compositional profile of the sample or significant differences in the processing, species, or environmental factors. The low carbohydrate content could make this sample particularly appealing for low-carb dietary applications or specialized nutritional products.

The obtained fiber content of 24.4 ± 4.9 g/100 g DW is relatively high compared to most values reported in the literature. It is higher than that found by Bhuylan et al. [50] at 11.51 ± 1.32 g/100 g, Chin et al. [52] at 19.08 ± 0.35 g/100 g, and Warnasooriya et al. [53] at 12.33 ± 0.08 g/100 g. It also greatly exceeds the low value of 2.1 g/100 g reported by Hong et al. [49] and 6.55 g/100 g by Kasmiati et al. [54].

The presence and concentration of free amino acids play a crucial role in determining the taste of seaweeds—such as their sweetness, sourness, and bitterness—and are also known to enhance the umami flavor [55]. Free amino acids can be classified into four categories based on their impact on taste perception. Sweet-tasting amino acids include threonine, serine, glycine, alanine, and proline. Aspartic acid and glutamic acid are associated with the umami taste. Bitter-tasting amino acids comprise valine, methionine, isoleucine, leucine, phenylalanine, histidine, arginine, and tryptophan. Lastly, tyrosine and lysine are considered tasteless [56]. *C. cylindracea* contains amino acids from all three taste categories—sweet, bitter, and umami—contributing to its strong and complex flavor profile. The intensity of the umami taste is commonly expressed as the Equivalent Umami Concentration (EUC), based on the levels of free amino acids such as glutamic acid and aspartic acid, and some nucleotide values (e.g., IMP, GMP, and AMP). This value indicates the amount of monosodium glutamate (MSG) equivalents per 100 g of DW [57,58]. Monosodium glutamate (MSG) is a widely used flavor enhancer in the food industry [59]. Despite its popularity, MSG remains controversial, with ongoing debate about its safety. Reports of adverse reactions to monosodium glutamate (MSG)—including headaches, flushing, and paresthesia, collectively referred to as the “MSG symptom complex”—have been documented. However, the scientific evidence establishing a causal relationship between MSG consumption and these symptoms remains limited and inconclusive. Consequently, interest has grown in identifying natural flavor enhancers that may represent safer or more desirable alternatives [59,60]. *C. cylindracea*, therefore, holds potential for application as a natural flavor enhancer in food formulations and prepared meals.

Protein levels in edible *Caulerpa* species vary widely. De Gaillande et al. [44] reported a range of 0.6% to 20.8% dry matter across various species, while *C. racemosa* showed an even broader range—from 0.64% to 56.47% of dry matter [4]. Such variability in the protein content can be attributed to multiple external factors, including the water temperature, seasonal fluctuations, and geographic location [4]. The protein quality depends on the presence and proportion of essential amino acids [44]. The amino acid profile of *Caulerpa cylindracea* is comparable to that of other edible *Caulerpa* species, with certain amino acids such as lysine showing slightly higher levels, while others like glutamic acid are somewhat lower [44].

*C. cylindracea* exhibits a distinctive fatty acid (FA) profile that is consistent with other species within the *Caulerpa* genus, particularly *C. racemosa*, yet it also presents several notable features [4]. The lipid fraction is primarily composed of polyunsaturated fatty acids (PUFAs), including omega-3 (n-3) fatty acids, which are known for their beneficial effects on cardiovascular and neurological health [61,62]. Among these, α-linolenic acid (ALA, C18:3n-3) was the most abundant omega-3 fatty acid, followed by eicosapentaenoic acid (EPA, C20:5n3). These omega-3 PUFAs are known for their anti-inflammatory and cardioprotective properties [63], highlighting the nutritional potential of this seaweed despite its overall low-fat content. The ratio of n-6 to n-3 fatty acids in *C. cylindracea* was within the recommended range for human nutrition [64], supporting its potential as a functional food ingredient. Saturated fatty acids (SFAs) were present at lower levels, with a slightly higher proportion of palmitic acid (C16:0), which is typical of many marine algae [23,44]. Palmitic acid was also recorded as the most dominant fatty acid component by Blažina et al. [27], who studied the same species from the same region. The low levels of trans-fatty acids also support the nutritional value of this seaweed. Although not a significant source of total lipids, this seaweed contains a lipid fraction enriched in bioactive unsaturated fatty acids, particularly omega-3 polyunsaturated fatty acids, thereby supporting its potential application in the development of functional foods and health-promoting dietary strategies [65]. Further research into seasonal and geographical variation in the FA content is recommended, as these factors significantly influence the lipid composition in macroalgae [27,66].

Generally, seaweeds contain 10 to 20 times more minerals compared to land plants. They accumulate these minerals from seawater, making them rich in both macro-elements and trace elements. The ash content in seaweeds, which reflects their mineral content, is typically high, ranging from 20 to 50% of DW [67]. In *Caulerpa* species, ash values can reach up to 55% [44]. *C. cylindracea* has registered an ash content of 46.1 g/100 g DW, placing it on the higher end of this range. Seaweeds accumulate minerals from their environment, but it is important to keep in mind that they can also absorb harmful metals, which may compromise their safety for human consumption [67]. Interestingly, among the 23 edible green seaweeds analyzed, data for Cu, Mn, and Zn were found only in *Caulerpa* spp. and *Ulva clathrate* [67]. Compared to *C. racemosa*, *C. cylindracea* exhibited comparable or higher levels of Fe, Mg, Ca, Mn, Cu, Zn, K, and Na. The elevated sodium content and its associated health implications have been addressed in the preceding section. Notably, the iron content in *C. cylindracea* is significantly higher than the average values reported for *C. racemosa*. Iron intake is essential for the body, especially during periods of growth and pregnancy, as a deficiency can lead to anemia [68]. Calcium, zinc, and copper levels in *C. cylindracea* are comparable to the average values found in *C. racemosa*. Zinc is an essential micronutrient involved in the function of over 300 enzymes and 1000 transcription factors [69], and it plays a critical role in the body’s defense against pathogens [70]. Mg and Ca are essential for bone and teeth health. Magnesium supports around 300 enzyme systems, while calcium contributes to heartbeat regulation, muscle contraction, and the activation of insulin and the thyroid hormone calcitonin. Copper is important for hemoglobin synthesis, which is essential for maintaining connective tissue strength, facilitating vitamin C oxidation, and promoting hemoglobin synthesis [67]. *C. cylindracea* shows significantly higher manganese levels compared to the average values for *C. racemosa*. Manganese plays a role in the metabolism of proteins, lipids, and carbohydrates, and is essential for immune function, blood sugar regulation, and cellular energy production [71]. A high dietary intake of manganese is generally not harmful in individuals with normal kidney function, as hypermanganesemia is unlikely to result from food sources alone [67]. As stated before, the presence of metals that are dangerous to human health has not been detected in *C. cylindracea*.

Plants typically cannot synthesize vitamin B12, but it is abundant in seaweeds [72]. It has been reported that *Caulerpa* species, like other edible algae such as nori, contain substantial amounts of vitamin B12 and may help prevent deficiencies in vegetarian and vegan diets [73]. *C. cylindracea* has been shown to contain a significant amount of vitamin B12, making it a potentially valuable addition to a vegan diet. According to the European Food Safety Authority (EFSA), consuming 1 g of dry seaweed satisfies nearly 10% of the recommended daily intake. This means that 10 g of dry seaweed covers almost the entire daily need for vitamin B12 [74]. There are limited comparative data regarding the vitamin content available for *C. racemosa*. However, when compared with other species, *C. cylindracea* exhibits notably high levels of both vitamin B12 and vitamin E [44]. The vitamin B12 content exceeds that reported for *C. racemosa* var. *turbinata*, and the vitamin E content in *C. cylindracea* is particularly remarkable, reaching 53 mg/100 g DW. This is also higher than the values reported for *Caulerpa lentillifera* [25]. This unusually elevated level may reflect both the species’ biochemical plasticity as an invasive alga and the winter sampling conditions, where a low temperature, fluctuating light, and increased PUFA content likely enhanced the need for lipid protection, thereby promoting high vitamin E accumulation [75,76]. The high vitamin E content provides strong antioxidant effects that may boost resistance to disease and oxidative stress. Antioxidants play a crucial role in protecting against various health conditions, including chronic inflammation, atherosclerosis, cancer, cardiovascular disease, and the ageing process [77]. In this regard, the neuroprotective effect of vitamin E has been demonstrated through its ability to reduce oxidative stress, which in turn results in a lower number of p53-positive brain cells [78]. Furthermore, vitamin E plays an important role in cellular protection and has been shown to contribute to chemoprevention [79]. The consistent consumption of *C. cylindracea* may serve as a natural source of antioxidants, contributing to overall health and well-being. The high content of vitamin E and salt could also be valuable as natural preservatives in the food industry. Vitamin E could help prevent lipid oxidation and extend the shelf life, while salt acts as an effective agent for inhibiting microbial growth. In addition, *C. cylindracea* may impart flavor-enhancing properties, particularly in seafood products, thereby improving their sensory attributes. Together, these characteristics may enhance stability, safety, and palatability, offering a natural alternative to synthetic additives.

The comprehensive evaluation of both the benefits and risks associated with Caulerpa as a food source is critical to inform consumer choices and to explore sustainable strategies for the utilization of *C. cylindracea* as a resource [35]. *Caulerpa* species are known to contain secondary metabolites (caulerpin, caulerpicin, and caulerpenyne), which, despite their positive pharmacological effects, may also pose toxicity risks [29]. Caulerpin and monomethyl caulerpinate have been isolated in *C. cylindracea* [80]. A study by Vidal et al. [81] demonstrated that caulerpin and caulerpicin are non-toxic in mice following oral and intraperitoneal administration. A recent study on *C. racemosa* ethanolic extract found no adverse effects on mice’s vital organs during a 28-day trial [82]. Moreover, *C. cylindracea* extract demonstrated the ability to repair mucosal damage caused by indomethacin in male *Rattus norvegicus* rats [83]. Further studies are necessary to shed light on this aspect or to determine the dose above which these chemicals could pose toxicity, as seen, for instance, in the use of nutmeg [84,85]. Generally, there is a significant knowledge gap regarding the in vivo digestion of seaweeds and their products, including nutrient assimilation and bioavailability. Interactions between seaweed components and other food constituents may affect the bioavailability of essential nutrients and bioactive agents [29].

## 5. Conclusions

*C. cylindracea* exhibits promising nutritional characteristics, including a high content of vitamin B12—an important nutrient for vegan diets—and notable levels of vitamin E, alongside substantial mineral and amino acid profiles. While its high salt content necessitates a desalination process for use in larger quantities, this inherent saltiness could also serve as a natural preservative in food applications. The significantly lower carbohydrate content and relatively high fiber level suggest that the analyzed sample may offer nutritional benefits suitable for low-carbohydrate, high-fiber diets. Overall, *C. cylindracea* holds potential as a valuable ingredient both for its nutritional benefits and its functional properties in the food industry. Further studies are needed to assess the potential toxicity and antinutritional factors of *Caulerpa cylindracea*, as well as to investigate the seasonal dynamics of its nutritional profile and biomass fluctuations over time.

## Figures and Tables

**Table 1:** Proximate composition (g/100 g DW), energy value (kcal), and salt content (g/100 g DW) of *Caulerpa cylindracea*. Each value in the table is represented as average ± SD (n = 3).

Parameter	Values
Energy value	154
Proteins	11.8 ± 0.9
Carbohydrates	11.6
Sugars	<0.1
Fibers	24.4 ± 4.9
Fats	1.3 ± 0.5
Saturated fatty acids	0.4 ± 0.1
Moisture	4.8 ± 0.5
Ash	46.1 ± 2.77
Salt (NaCl)	33.8 ± 6.8

**Table 2:** Amino acid composition (mg/100 g DW) of *Caulerpa cylindracea*. Each value in the table is represented as average ± SD (n = 3).

Amino Acid	Values
Essential amino acids	
HIS	160 ± 30
ISO	380 ± 60
LEU	610 ± 100
LYS	480 ± 80
MET	170 ± 30
PHE	430 ± 70
THR	450 ± 70
TRP	142 ± 20
VAL	540 ± 90
Non-essential amino acids	
TYR	360 ± 60
ALA	520 ± 80
PRO	420 ± 70
CYS	200 ± 30
ASP	270 ± 40
GLU	630 ± 100
SER	440 ± 70
GLY	540 ± 90
ARG	480 ± 80

**Table 3:** Mineral composition (mg/kg DW) of *Caulerpa cylindracea*. Each value in the table is represented as average ± SD (n = 3) (non-detected elements are indicated as ‘nd’).

Element	Values
Al	629 ± 92
B	29.3 ± 3.6
Ca	9010 ± 1270
Cd	nd
Co	nd
Cr	nd
Cu	2.36 ± 0.40
Fe	428 ± 55
K	1220 ± 1600
Li	7.34 ± 0.44
Mg	10,700 ± 1100
Mn	164 ± 21
Mo	nd
Na	over
Ni	nd
P	785 ± 87
Pb	Nd (mg/kg)
S	19,300 ± 1700
Se	nd
Si	28.7 ± 5.2
Zn	19.3 ± 2.4

**Table 4:** Vitamin composition (µg/g DW) of *Caulerpa cylindracea*. Each value in the table is represented as average ± SD (n = 3).

Vitamin	Values
Biotin (B7)	0.031 ± 0.011
Folic acid (B9)	0.060 ± 0.039
Niacinamide (B3)	1.53 ± 0.05
Nicotinic acid (B3)	5.14 ± 0.09
Pantothenic acid (B5)	1.43 ± 0.06
Pyridoxal (B6)	0.59 ± 0.02
Pyridoxine (B6)	0.092 ± 0.004
Riboflavin (B2)	0.996 ± 0.0028
Vitamin E (as alpha-tocopherol)	525 ± 43
Cyanocobalamin (B12)	0.39 ± 0.08

**Table 5:** Fatty acid composition (mg/g DW) of *Caulerpa cylindracea*. Each value in the table is represented as average ± SD (n = 3).

Fatty Acid	Composition
Saturated fatty acids	
C4:0 (Butyric)	<1 (1 ± 1)
C6:0 (Caproic)	<1 (1 ± 1)
C8:0 (Octanoic)	<1 (1 ± 1)
C10:0 (Decanoic)	<1 (1 ± 1)
C11:0 (Undecanoic)	<1 (1 ± 1)
C12:0 (Lauric)	<1 (1 ± 1)
C13:0 (Tridecanoic)	<1 (1 ± 1)
C14:0 (Myristic)	1 (1 ± 1)
C15:0 (Pentadecanoic)	<1 (1 ± 1)
C16:0 (Palmitic)	3 (1 ± 1)
C17:0 (Heptadecanoic)	<1 (1 ± 1)
C18:0 (Stearic)	<1 (1 ± 1)
C20:0 (Arachidic)	<1 (1 ± 1)
C21:0 (Heneicosanoic)	<1 (1 ± 1)
C22:0 (Behenic)	<1 (1 ± 1)
C23:0 (Tricosanoic)	<1 (1 ± 1)
C24:0 (Lignoceric)	<1 (1 ± 1)
Total saturated fatty acids (sAFA)	4 (1 ± 1)
Monounsaturated fatty acids	
C14:1 (Myristoleic)	<1 (1 ± 1)
C15:1 (Ginkgolic)	<1 (1 ± 1)
C16:1n7 (Palmitoleic)	<1 (1 ± 1)
C17:1 (Heptadecaenoic)	<1 (1 ± 1)
C18:1n9 (trans-Elaidic)	<1 (1 ± 1)
C18:1n9 (Oleic)	2 (1 ± 1)
C18:1n7 (Vaccenic)	<1 (1 ± 1)
C20:1 (Eicosenoic)	<1 (1 ± 1)
C22:1n11 (Gadoleic)	<1 (1 ± 1)
C22:1n9 (Erucic)	<1 (1 ± 1)
C24:1n9 (Nervonic)	<1 (1 ± 1)
Total monounsaturated fatty acids (MUFA)	3 (1 ± 1)
Polyunsaturated fatty acids	
C16:2n4 (Hexadecadienoic)	<1 (1 ± 1)
C16:3n4 (Hexadecatrienoic)	<1 (1 ± 1)
C18:2n6 (trans-Linoleic)	<1 (1 ± 1)
C18:2n6 (Linoleic)	<1 (1 ± 1)
C18:3n6 (γ-Linolenic)	<1 (1 ± 1)
C18:3n4 (Octadecatrienoic)	<1 (1 ± 1)
C18:3n-3 (α-Linolenic, ALA)	2 (1 ± 1)
C18:4n3 (Stearidonic)	<1 (1 ± 1)
C20:2n6 (Eicosadienoic)	<1 (1 ± 1)
C20:3n6 (Dihomo-γ-linolenic, DGLA)	<1 (1 ± 1)
C20:3n3 (Eicosatrienoic)	<1 (1 ± 1)
C20:4n6 (Arachidonic)	1 (1 ± 1)
C22:2n6 (Docosadienoic)	<1 (1 ± 1)
C20:4n3 (Eicosatetraenoic, ETA)	<1 (1 ± 1)
C20:5n3 (Eicosapentaenoic)	1 (1 ± 1)
C22:5n3 (Docosapentaenoic, DPA)	<1 (1 ± 1)
C22:6n3 (Docosahexanoic)	<1 (1 ± 1)
Total polyunsaturated fatty acids (PUFA)	5 (1 ± 1)
Other fatty acids	1 (1 ± 1)
Total Omega-3 fatty acids	3 (1 ± 1)
Total Omega-6 fatty acids	1 (1 ± 1)
Total Omega-9 fatty acids	2 (1 ± 1)
Total trans-fatty acids	<1 (1 ± 1)

## Data Availability

The original contributions presented in the study are included in the article/Supplementary Material, further inquiries can be directed to the corresponding author.

## Supplementary Materials

The following supporting information can be downloaded at: https://www.mdpi.com/article/10.3390/foods14183208/s1, Table S1: Limits of detection and quantification for mineral analysis.
