# Supplementary material for: Caulerpa cylindracea: First Insight into Its Nutritional Potential
Source: Foods. 2025 Sep 15;14(18):3208. doi: 10.3390/foods14183208 (PMC12469415; doi:10.3390/foods14183208)
Supplement: Supplementary file 1 [file foods-14-03208-s001.zip › foods-3821662-supplementary.pdf]

foods MDPI

Article

Caulerpa cylindracea: first insight into its nutritional potential

Neven Iveša [1], Ines Kovačić [1], Moira Buršić [1], Nikola Major [2], Igor Palčić[2], Smiljana Goreta Ban[2], Zoran Užila[2], and Gioconda Millotti [1,*]

[1] Faculty for Natural Sciences, Juraj Dobrila University of Pula, 52100 Pula, Croatia; neven.ivesa@unipu.hr (N.I.); ines.kovacic@unipu.hr (I.K.); moira.bursic@unipu.hr (M.B.); gmillotti@unipu.hr (G.M.)
[2] Department of Agriculture and Nutrition, Institute of Agriculture and Tourism, Carlo Hugues 8,52440 Poreč, Croatia; nikola@iptpo.hr (N.M.); palcic@iptpo.hr (I.P.); smilja@iptpo.hr (S.G.B.), zoran@iptpo.hr (Z.U.)
* Correspondence: gmillotti@unipu.hr

SUPPLEMENTARY MATERIAL

Table S1. Limits of detection and quantification for mineral analysis.

Element	Limit of detection (mg/L)	Limit of quantification (mg/L)
Al	0.0010269	0.003423
B	8.99E-04	0.0029981
Ca	0.0091969	0.0306562
Cd	1.53E-04	5.10E-04
Co	1.48E-04	4.95E-04
Cr	9.46E-04	0.0031533
Cu	0.0018606	0.006202
Fe	8.00E-04	0.0026679
K	0.0020678	0.0068928
Li	0.0017154	0.0057181
Mg	0.0038251	0.0127502
Mn	3.44E-04	0.001146
Mo	3.02E-04	0.001008
Na	0.0024149	0.0080495
Ni	3.74E-04	0.0012475
P	0.0110098	0.0366993
Pb	0.0028317	0.0094388
S	0.0019788	0.0065961
Se	0.0107734	0.0359114
Si	0.0536564	0.1788548
Zn	0.0088421	0.0294736
